# Individual versus general structured feedback to improve agreement in grant peer review: a randomized controlled trial

**DOI:** 10.1186/s41073-021-00115-5

**Published:** 2021-09-30

**Authors:** Jan-Ole Hesselberg, Knut Inge Fostervold, Pål Ulleberg, Ida Svege

**Affiliations:** 1 Department of Psychology, University of Oslo, Oslo, Norway; 2 Faculty of Health Sciences, Oslo Metropolitan University, Oslo, Norway

**Keywords:** Peer review, Inter-rater agreement, Funding, Reliability, Feedback, Training

## Abstract

**Background:**

Vast sums are distributed based on grant peer review, but studies show that interrater reliability is often low. In this study, we tested the effect of receiving two short individual feedback reports compared to one short general feedback report on the agreement between reviewers.

**Methods:**

A total of 42 reviewers at the Norwegian Foundation Dam were randomly assigned to receive either a general feedback report or an individual feedback report. The general feedback group received one report before the start of the reviews that contained general information about the previous call in which the reviewers participated. In the individual feedback group, the reviewers received two reports, one before the review period (based on the previous call) and one during the period (based on the current call). In the individual feedback group, the reviewers were presented with detailed information on their scoring compared with the review committee as a whole, both before and during the review period. The main outcomes were the proportion of agreement in the eligibility assessment and the average difference in scores between pairs of reviewers assessing the same proposal. The outcomes were measured in 2017 and after the feedback was provided in 2018.

**Results:**

A total of 2398 paired reviews were included in the analysis. There was a significant difference between the two groups in the proportion of absolute agreement on whether the proposal was eligible for the funding programme, with the general feedback group demonstrating a higher rate of agreement. There was no difference between the two groups in terms of the average score difference. However, the agreement regarding the proposal score remained critically low for both groups.

**Conclusions:**

We did not observe changes in proposal score agreement between 2017 and 2018 in reviewers receiving different feedback. The low levels of agreement remain a major concern in grant peer review, and research to identify contributing factors as well as the development and testing of interventions to increase agreement rates are still needed.

**Trial registration:**

The study was preregistered at OSF.io/n4fq3.

**Supplementary Information:**

The online version contains supplementary material available at 10.1186/s41073-021-00115-5.

## Background

Worldwide, vast sums are distributed through grants that use peer review processes. Despite this, evidence of the ability of peer review to identify the “best” future projects – however defined – seems sparse. The latest Cochrane review of peer review to improve the quality of grant proposals concludes that “there is little empirical evidence on the effects of grant-giving peer review” [1], and in the latest review of grant peer review, Guthrie et al. [2] conclude that there is “fairly clear evidence that peer review is, at best, a weak predictor of future research performance”. Because funding decisions relying on peer review have a considerable impact on how public funds are spent, the trajectories of a field’s knowledge base and the careers of researchers, the lack of predictive value is a concern.

One factor that is likely to contribute to poor predictive value is low levels of agreement between reviewers assessing the same proposal. Several studies have shown that grant reviews have very low levels of agreement. A study of nine different programmes at the National Science Foundation found average rating intraclass correlations (ICCs) between 0.18 and 0.37 [3], and a study of 23,414 ratings in six different research areas at the Austrian Science Fund found average rating ICCs between 0.38 and 0.55 [4]. Some disagreement must be expected due to differences in the reviewers’ background, opinions and perspectives, and disagreement resulting from these sources might even be desirable. This point has been made by Reinhart (2009) who writes that “Disagreements between reviewers are desirable if they are the result of different points of view (..) or differential emphasis on quality criteria” [5]. Similarly, Bailar (1991) makes a valid point when highlighting that “editors and grant managers choose (and should choose) reviewers for their different, complementary expertise”, and hence high levels of agreement might even be a cause of worry [6]. Still, there are degrees of agreement and if the final decisions rests on reviews without reasonable levels of agreement and the disagreement is not compensated for by adding enough reviewers, the overall decision-making process and funding decisions will become unreliable [7]. In their review of the literature on grant peer review, Marsh et al. [8] conclude that “interrater reliability estimates are not adequate, falling well below acceptable levels”. These findings are in line with findings in journal peer review. In a meta-analysis of inter-rater reliability in journal peer review, Bornmann et al. [9] found mean levels of ICC of 0.34 and Cohen’s kappa of 0.17.

One reason for the low agreement levels might be different scoring styles and different interpretations of the scoring scale among reviewers. Without a common understanding of how to interpret and use the scale, differences in scoring among reviewers easily occur in the evaluation process. Reviewer training delivered by funders is one way to increase the level of shared understanding, but studies show that such initiatives are lacking. A survey of 57 international public and private grant-giving organizations found that only 9% of the reviewers had received any formal training and that “64 % of the reviewers said they would be interested in receiving training if funding organizations provided it” [10].

In funding programmes where a set of reviewers are engaged to review many proposals in multiple calls for proposals over some time, simple feedback on how the reviewers use the scale compared to other reviewers might be a cost-efficient way to calibrate reviewers’ use of the scale. A lenient reviewer with a tendency to use only the top scores might employ feedback in a way that makes him or her use the lower scores more frequently and distribute them more in accordance with the score distribution of the other reviewers. Reduced variability in the distribution of scores between reviewers will increase the likelihood of absolute agreement (similar scores by reviewers). Since this feedback might also contribute to larger variability in the distribution of scores within reviewers and thereby change the individual reviewer’s ranking of the proposals, it might also increase relative agreement.

Research on the effects of reviewer training on any outcome, let alone reviewer feedback on agreement in grant peer review, is sparse. The most up-to-date systematic review of the effect of training in journal peer review included only five studies [11]. Based on the five qualified studies, the review concluded that “training did not improve the quality of the peer review report”. Most studies identified in this review and in other training trials [12, 13] test the effect of structured training or mentoring sessions, and the outcomes seldom include interrater reliability or agreement as the outcome measure. In addition, most of the studies focus on journal peer review rather than grant peer review. To our knowledge, the study by Sattler et al. [12] is the only study to test the effect of training on interrater reliability in grant peer review. They found that interrater reliability, as measured by intraclass correlation, was significantly higher in the group receiving an online course (0.89) compared to a group just receiving written information (0.61). However, the participants did not review proposals; instead, they “received criteria consistent with specific rating scale values and were asked to assign ratings that were consistent with those criteria”.

The objective of this study was to evaluate the effect on the agreement between reviewers receiving a simple individual feedback report compared to a general feedback report. Our preregistered main hypothesis was that agreement, either in the assessment of eligibility or the scoring of proposals, would differ between reviewers who received individual feedback reports on their scoring and reviewers who received general and nonspecific feedback reports. Our secondary hypothesis was that the perceived usefulness of the feedback report would differ between reviewers who received an individual feedback report and those who received a general feedback report.

## Methods

### Trial design

We conducted a randomized controlled design with two parallel groups. One group of reviewers received an individual feedback report, and the other group received a general feedback report. The study was preregistered at Open Science Framework (osf.io/n4fq3) [14].

### Participants and setting

The participants included in this study comprise all reviewers who served in the funding programme Health (“Helse”) at the Norwegian Foundation Dam in both 2017 and 2018 (*n* = 48).

The Health program has two calls for proposals every year, and each call has two different review committees (Health spring and Health fall), with 24 reviewers in each. In 2017, 1197 project proposals were submitted (639 in spring, 558 in fall). The following year, in 2018, 1043 were submitted (512 in spring, 531 in fall). Proposals had to deal with “physical and mental health, coping, quality of life or social participation”; and using the Health Research Classification System (HRCS) [15] most were classified as covering topics of “Mental health”, or “General health relevance”. All proposals had to be submitted by NGOs and not all were research related. All proposals were submitted individually, had to be written in Norwegian and the project proposal had a maximum length of ten pages, including references. There was no limit to how many proposals could be submitted, and no applicants were excluded based on previous grants or rejections. Review scores and comments were only shared with applicants by request, and the applicant could not appeal the funding decision.

Each proposal was assigned to three reviewers by an algorithm that matched the topic of the proposal (defined by the HRCS health categories) to the profile of the 24 reviewers in the call. Each proposal was then evaluated in two rounds through an electronic review form. The first round consisted of two steps. In the first step, matched reviewers answered “yes” or “no” to the question, “Is the proposal eligible for the programme?”. In the second step, if the reviewer considered the proposal to be eligible, the reviewer then scored the quality of the proposal using 13 criteria, including originality, feasibility, dissemination of results and user involvement. The reviewer provided an overall proposal score “on a scale from one to ten, where ten is the best”. Reviewers were not asked to make a recommendation for funding. Three reviewers worked independently of each other within a time frame of 35 days for first round reviews. If a proposal was considered ineligible by two or all three reviewers, it was rejected at this stage. Additionally, based on the average scores of reviews from the first round, in 2017 and 2018, 12% were funded immediately, 62% rejected, and 25% proceeded to the second round. In the second round the proposals were reviewed by a second set of three reviewers. However, only first round reviews were included in this study. Overall, acceptance rates were 23% in 2017, and 26% in 2018, with the maximum funding amount per project was NOK 3 million, and the average amount was NOK 752,000. All reviewers were Norwegian, and they declared conflicts of interest in relation to the proposals. The reviewers were paid NOK 220 per review and in 2017 and 2018 the average reviewer conducted 75 and 65 reviews, respectively.

All reviewers signed an informed consent form (see osf.io/pkdtv) before study enrolment, and none received additional payment to participate in the study. Baseline characteristics of the participants included age, gender and years of experience as a reviewer for the foundation.

### Interventions

Two interventions were delivered prior to and during the 2018 review period: general feedback and individual feedback. Both were delivered electronically in the form of one-page reports that were designed and generated in a Microsoft® Excel workbook. The participants were only informed that they would receive different forms of feedback, not that one of the feedback reports were designed as a control intervention (see consent form at osf.io/pkdtv).

**Fig. 1:**
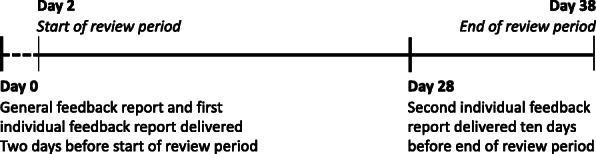
Timeline of intervention delivery in the review periods of 2018

The general feedback was designed as a control intervention. The usual process was not used as a control condition for two reasons. First, the usual process was unstructured written or verbal feedback only if the reviewers asked for it and we suspected this would result in most of the reviewers in the control condition asking for feedback when realising they were in the control condition. Second, we wanted to prevent potential variation in observer effects between the two groups. The general feedback was designed in a way to provide the reviewers with information they were likely to find interesting, but that did not provide them with specific information on their individual reviews. It consisted of one simple report containing information only on the total number of proposals rated by the review committee as a whole in the previous call in 2017 and a line chart of the distribution of the committee’s review scores. The reviewers in the general feedback group received the report only once, 2 days before the start of the review period (Fig. 1).

The individual feedback intervention consisted of two reports with detailed information on each recipient’s reviews compared to the reviews by the committee as a whole. The first individual feedback report was delivered 2 days before the start of the review period and was based on the reviews from the previous call in 2017. The second was delivered 10 days before the end of the review period and was based on the reviews conducted to date in the ongoing call (Fig. 1). On average the reviewers completed 70% (range 29 to 100%) of the assigned reviews before receiving the second report. The rationale for the second report was to give the reviewers feedback on a set of reviews that could still be adjusted.

Both individual feedback reports comprised information on the reviewer’s assessment of eligibility, scoring of the proposals, and level of agreement with other reviewers of the same proposals. This information was presented in tables, figures and text. The report included a table showing the proportion of proposals the reviewer rated as ineligible and a table showing the reviewer’s average review scores compared to the review committee as a whole. In addition, the report contained a graph showing the distribution of the reviewer’s scores compared to the score distribution of the committee as a whole. The tables and charts were accompanied by standardized text segments describing the same information. Apart from the different data basis for the two reports, they differed only in that the first included information on how often the reviewer agreed with at least one of the other reviewers, while the second report did not. Translated versions of the two reports can be viewed at dam.no/feedback.

The feedback reports were designed primarily by two of the authors, JOH and IS, in close collaboration with the administration of the foundation and selected reviewers. The feedback reports were not used prior to the 2018 calls.

### Outcomes

#### Main outcomes: agreement

For the main outcome measures the level of agreement based on pairs of reviews of the same proposal within the same intervention group (individual or general) was analyzed. Paired reviews were used because 1) the intervention (individual feedback report) was intended to be used for all reviewers in a committee, and 2) only about one third of the data could have been used if we had limited the analyses to proposals where all three reviewers were in the same group.

##### Agreement in the review of eligibility

The first main outcome was the agreement in the scoring of eligibility in round one between pairs of reviewers within the same intervention group who had reviewed the same proposal, categorized as “agree” or “disagree”. In the following, this is referred to as “eligibility agreement”.

##### Agreement in the review of the quality of the proposals

The second main outcome was the average absolute difference in overall proposal score in round one between pairs of reviewers within the same intervention group who had reviewed the same proposal. In the following, this is referred to as the “average absolute difference”. Traditionally, both kappa statistics and different versions of Intraclass Correlation Coefficients (ICC) have been extensively applied in studies measuring reviewer agreement [3, 4, 9]. In addition, the average deviation (AD) index, has been used. Seeber et al. (2021) used the AD index in an analysis of agreement in a multilevel analysis of 52,488 proposals and describes it as “the extent to which a reviewer’s evaluation of a proposal deviates from the mean of the individual scores of the proposal.” [16]. Pina et al. used the same strategy in their analysis of more than 70,000 Marie Curie proposals and argue that benefits of the AD index is that it can be calculated at the both the reviewer and proposal level and that it “does not require the specification of null distribution and returns values in the units of the original scale, ( …) making its interpretation easier and more pragmatic” [17]. In the present study, the average absolute difference between paired reviews was calculated. Since only two scores were used, the average absolute difference will always be double the AD index and will provide the reader with the exact distance between the reviewers, likely making the interpretation even easier.

#### Secondary outcomes: perceived usefulness

The secondary outcome measure was the reviewers’ perceived usefulness of the report. All reviewers received a web-based survey with two questions regarding the perceived usefulness of the interventions. They answered the following questions:

- “To what degree did you find the feedback you received useful?” on a five-point Likert scale (To a very small degree, To a small degree, To some degree, To a large degree or To a very large degree).
- “If you were offered this feedback next time, would you want it?” by choosing “yes”, “no” or “I don’t know”.

#### Changes to outcomes

In the study preregistration, agreement regarding the proposal score was defined as the “difference in total review score of 0 or 1”. Consequently, the planned main outcome measure was dichotomous (“agree” and “disagree”). However, the average absolute difference provided a continuous, more fine-grained measure of agreement, and it was therefore chosen as the main outcome in the study. Both outcomes were derived from the reviewer’s quality scoring, and the predefined outcome measure was used in the sensitivity analyses.

### Sample size

All eligible reviewers were included as participants. Hence, no power analysis was conducted prior to inclusion.

### Randomization

The participants were randomized in two rounds, one for each committee. The randomization was done by JOH in Microsoft® Excel for Mac version 16.10 by using the rand.between function and drawing a random number between 1 and 10,000 for each participant. The half with the lowest numbers were assigned to the general feedback group, the half with the highest numbers were assigned to the individual feedback group. The participants in the spring committee were randomized first. In the randomization of the fall committee, the reviewers who also served on the spring committees were assigned to the same group as they were in the spring. All feedback reports for both groups were sent to participants by JOH by e-mail.

### Blinding

This was a double-blinded trial. The reviewers were not informed about the differences between the two interventions provided. They were only told that they would receive one of two feedback reports and did not know what information the other group received in their report. The personnel who interacted directly with the reviewers were not aware of the assigned interventions. In addition, the author who conducted the initial statistical analysis (IS) was blinded and was provided with anonymized data sets.

### Statistical methods

#### Main outcome: agreement

To examine whether the intervention had an effect on eligibility agreement and the difference in scores between reviewers, linear mixed models (LMM) were used. To examine the effect of the intervention on eligibility agreement, we used a linear mixed binary logistic regression model, where reviewer’s ratings (0 = not eligible, 1 = eligible) were nested within proposals.

To examine the effect on score difference a linear mixed-effects regression model was used. This was preferred since three reviewers were nested within the same proposal, yielding at most three absolute difference scores for each proposal given that all reviewers found the proposal eligible. Thus, the reviewers’ absolute difference in scores was defined as level 1 in the model, and proposals were defined as level 2. The absolute difference in score was the dependent variable, and random intercepts for each proposal were included in the LMM. The covariance matrix of within-subject measurements was variance components. The time point (2017 vs. 2018) and group (individual feedback report vs. general feedback report) were included as dummy-coded fixed effects. We also included the interaction between group and time point as a fixed effect to estimate whether the change in the absolute difference score from 2017 to 2018 was dissimilar in the two groups. A significant interaction effect would indicate a significant effect of the individual feedback report. Because the absolute score differences were used, and hence all differences were positive, the distribution of the absolute differences deviated somewhat from a normality (skewness = 0.86, kurtosis = 0.56). In order to make the tests for statistical significance reliable, bootstrapped bias corrected confidence intervals (CI) based on 1000 samples were estimated. In addition, we calculated Gwet’s agreement coefficient 1 (AC1) for the eligibility agreement for the two groups. Gwet’s AC1 was used instead of the preplanned Cohen’s kappa because it is less sensitive to differences in prevalence and marginal probabilities [18]. The intraclass correlation was calculated for the average absolute difference. Since the raters were not the same for all subjects, we used the one-way random effects model, ICC (1, 2), for the average absolute difference.

The LMM analyses were deviations from the preregistered analysis plan. Initially, we planned to calculate the proportion of absolute agreement (for both eligibility and proposal score) and compare the groups using Fisher’s exact test. The LMM analysis provided a more fine-grained measure of agreement and the possibility to control for the interaction between group and time point. The preplanned analyses were also conducted and included as sensitivity analyses. Additionally, to assess the effects of differences in the scoring styles of reviewers we estimated a multilevel model where absolute differences were nested within reviewers instead of within proposals.

All analyses were performed according to the intention-to-treat principle. *P*-values below .05 were considered statistically significant.

#### Secondary outcome: perceived usefulness

We used the Mann-Whitney U test to compare the reviewers’ evaluation of the usefulness of the feedback report between the two groups, and the Fischer’s exact test to compare group differences in the response to the question “If you were offered this feedback the next time, would you want it?”

## Results

### Participant enrolment and characteristics

**Fig. 2:**
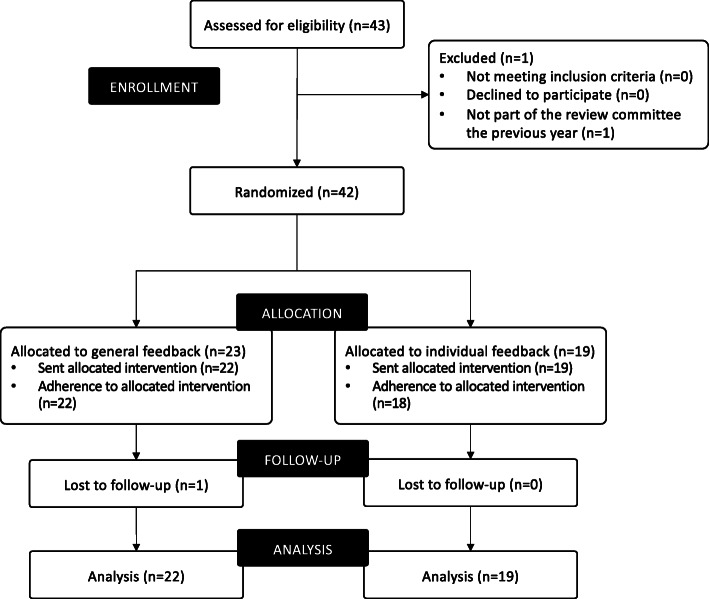
Study flowchart. Enrolment, randomization, and follow-up of study participants

The two review committees in the funding programme Health at the Norwegian Foundation Dam for the year 2018 consisted of 24 reviewers each. Five reviewers were members of both committees, leaving a total of 43 different reviewers to be assessed for eligibility (Fig. 2). One was excluded due to not being part of the review committee the previous year. The remaining 42 reviewers were included in the study and randomized to either the general feedback group (*n* = 23) or the individual feedback group (*n* = 19).

One participant in the general feedback group could not perform his reviews due to acute illness. Hence, he did not receive the allocated intervention. Follow-up data and data on compliance were retrieved for the remaining 41 participants, and none of them were excluded from the analyses. All participants in the general feedback group and 95% of the participants in the individual feedback group confirmed that they had received and read the feedback report.

Participant characteristics were similar in the two intervention groups (Table 1).

**Table 1:** Baseline characteristics of the study participants

Baseline characteristics	General feedback group (***n*** = 22)	Individual feedback group (***n*** = 19)	Total (***n*** = 41)
Age, years	58 ± 11,4	49 ± 9,4	54 ± 11,4
Women, count (%)	14 (64%)	9 (47%)	23 (56%)
Years of experience as reviewer for the foundation, median (inter quartile range)	1 (1,0 to 4,0)	1 (0,5 to 4,0)	1 (1,0 to 4,0)
Number of proposals submitted, count	–	–	1197
Average number of reviews per reviewer	74,0 ± 5,49	75,5 ± 5,11	74,7 ± 5,30
Number of proposals included in baseline analyses, count	511	409	920
Average number of reviews per reviewer included in analyses of the paired reviews	54,6 ± 7,17	45,7 ± 12,20	50,5 ± 10,70
Total number of paired reviews, count	715	545	1260
Average proposal score (1–10)	6,3 ± 1,93	5,7 ± 2,14	6,1 ± 2,05
Absolute differences^a^, count (%)	601 (84%)	434 (80%)	1035 (82%)
Average absolute difference	2,0 ± 1,54	2,2 ± 1,59	2,1 ± 1,56

Values are mean ± SD unless otherwise stated. ^a^Proposals where both reviewers in the paired review considered the proposal to be eligible and a score difference could be calculated

### Numbers analysed

A total of 2240 proposals were submitted, 1197 in 2017 and 1043 in 2018. After the removal of data related to the excluded reviewers and constructing pairs of the reviews, a total of 2398 paired reviews were analysed (Table 2). In 2038 (85%) of these cases, the two reviewers agreed that the proposal was eligible, and the average absolute difference could be calculated.

**Table 2:** Study dataset

	General feedback group (*n* = 22)			Individual feedback group (*n* = 19)		
Study dataset description	2017	2018	Total	2017	2018	Total
Total number of paired reviews	715	642	1357	545	496	1041
Number of proposals in the study	511	450	961	409	376	785
Number of paired reviews included in analyses of average absolute difference	601	594	1195	434	409	843
Number of paired reviews included in eligibility agreement analyses	715	642	1357	545	496	1041

### Outcomes and estimation

#### Main outcome: agreement in the review of eligibility

The results from the LMM analysis (Table 3) of eligibility agreement found no main effect of either time or group but a significant time×group interaction effect (*b* = 0.77, *p* = .006, *OR* = 2.17). The interaction effect indicated an increase in the proportion of eligibility agreement over time for the general feedback group only.

**Table 3:** Linear mixed binary logistic regression model analysis of eligibility agreement over time by group

	*b* (*se*)	*t*-value	*p*	*Odds Ratio* [95% CI]
Intercept	1.65 (0.13)	12.6	< .001	5.19 [4.02, 6.70]
Time^*a*^	0.08 (0.19)	0.43	.667	1.09 [0.75, 1.58]
Group^*b*^	0.19 (0.18)	1.05	.293	1.21 [0.85, 1.71]
Time×Group	0.77 (0.28)	2.74	.006	2.17 [1.25, 3.79]

^a^2017 = 0, 2018 = 1. ^b^Individual feedback group = 0, General feedback group = 1

**Fig. 3:**
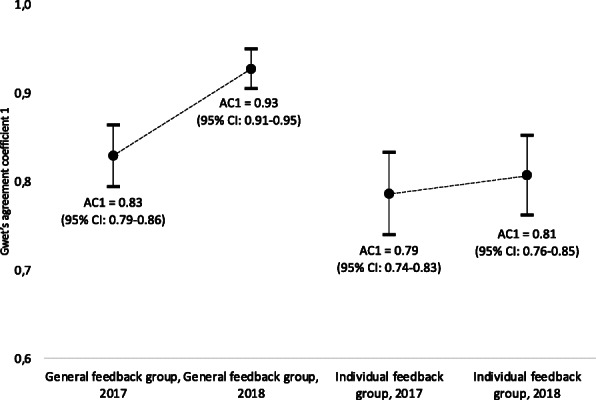
Gwet’s agreement coefficient 1 (AC1) in the general feedback and individual feedback groups in 2017 and 2018

Gwet’s AC1 in 2017 for the general feedback group and the individual feedback group was 0.829 (95% CI from 0.794 to 0.864) and 0.786 (95% CI from 0.762 to 0.852), respectively. In 2018, the values were 0.927 (95% CI from 0.905 to 0.950) for the general feedback group and 0.807 (95% CI from 0.762 to 0.852) for the individual feedback group (Fig. 3). The rate of absolute eligibility agreement ranged from 83 to 93% over both groups and both years (Table 4).

**Table 4:** Reviewer agreement and proposal scores for the Health program by the Norwegian Foundation Dam in 2017 and 2018

	General feedback group (*n* = 22)		Individual feedback group (*n* = 19)	
Variable	2017	2018	2017	2018
Total number of paired reviews	715	642	545	496
Eligibility agreement, count (%)	612 (86%)	599 (93%)	453 (83%)	417 (84%)
Absolute differences^a^, count (%)	601 (84%)	594 (93%)	434 (80%)	409 (82%)
Average proposal score (1–10)	6,3 ± 1,93	6,6 ± 1,85	5,7 ± 2,14	6,2 ± 1,97
Average absolute difference	2,0 ± 1,54	1,8 ± 1,47	2,2 ± 1,59	1,9 ± 1,48

Values are mean ± SD unless otherwise stated. ^a^Proposals where both reviewers in the paired review considered the proposal to be eligible and a score difference could be calculated. The numbers differ from “Eligibility agreement” because the latter includes both agreement regarding eligibility and ineligibility

#### Main outcome: agreement in the review of the quality of the proposals

The results from the LMM analysis (Table 5) showed that the time×group interaction effect was not significant, indicating an equal decrease in the absolute difference score over time for the individual feedback and the general feedback groups. There was an overall significant decrease in the absolute difference score from 2017 to 2018 (*b* = − 0.32, *p* = .004). In 2017, the reviewers later assigned to the general feedback group had a lower average absolute difference than those later receiving individual feedback (*b* = − 0.24, *p* = .020).

**Table 5:** Linear mixed regression model parameters for proposal scores in the Health program by the Norwegian Foundation Dam in 2017 and 2018

Model parameters	*b* (*se*)	*t*-value	*p*	95% CI^*c*^
Intercept	2.20 (0.08)	28.59	<.001	[2.05, 2.32]
Time^*a*^	−0.32 (0.11)	−2.92	.004	[−0.50, − 0.14]
Group^*b*^	− 0.24 (0.10)	−2.33	.020	[−0.38, − 0.11]
Time×Group	0.17 (0.15)	1.21	.228	[−0.05, 0.42]

^a^2017 = 0, 2018 = 1. ^b^Individual feedback group = 0, General feedback group = 1. ^c^Bias corrected bootstrapped confidence intervals based upon 1000 samples

**Fig. 4:**
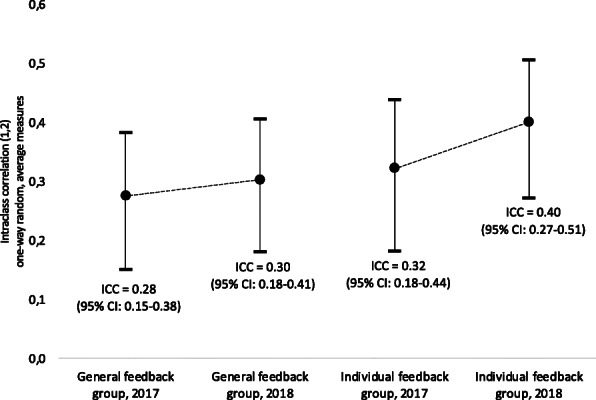
Intraclass correlation (1, 2) in the general feedback and individual feedback groups in 2017 and 2018

The ICC (1, 2) in 2017 for the general feedback group and individual feedback group was 0.276 (95% CI from 0.151 to 0.383) and 0.323 (95% CI from 0.182 to 0.439), respectively. In 2018, the values were 0.303 (95% CI from 0.181 to 0.406) for the general feedback group and 0.401 (95% CI from 0.272 to 0.506) for the individual feedback group (Fig. 4). The overall ICC [1, 3] for all three reviews for all submitted proposals, not just for the paired reviews included in the study, were 0.334 in 2017 and 0.428 in 2018 (Appendix Fig. 8). This is in line with the significant decrease in absolute score difference from 2017 to 2018 for the paired reviews (Table 5).

The mean proposal score in 2017 among those later receiving general feedback was 6.3 (95% CI from 6.19 to 6.49), while the mean score for those later receiving individual feedback group was 5.7 (95% CI from 5.52 to 5.90) (Table 4). In 2018 the mean score in the general feedback group was 6.6 (95% CI from 6.46 to 6.76), and the mean score in the individual feedback group was 6.2 (95% CI from 6.04 to 6.41).

#### Secondary outcome: perceived usefulness

Table 6 displays the perceived usefulness of the interventions. The results show that 95% (*n* = 18) in the individual feedback group and 68% (*n* = 15) in the general feedback group responded to the question “To what degree did you find the feedback you received useful?” after finishing the reviews. A Mann Whitney U test showed that there was no significant difference between the groups (*p* = 0.442). In addition, 80% in general feedback group and 94% in the individual feedback group answered “yes” to the question “If you were offered this feedback next time, would you want it?”. A Fischer’s exact test showed there was no significant difference between the groups (*p* = 0.382).

**Table 6:** Perceived usefulness of the interventions

	General feedback group (*n* = 15)	Individual feedback group (*n* = 18)	*p*
“To what degree did you find the feedback you received useful?”, mean score (scale 1–5)^a^	3,5 ± 0,74	3,7 ± 0,75	.442
“If you were offered this feedback next time, would you want it?”, number of “yes”/“no”/“I don’t know”	12/1/2	17/0/1	.382

^a^The question was answered on a five-point Likert scale (To a very small degree, To a small degree, To some degree, To a large degree or To a very large degree) and coded from 1 (To a small degree) to 5 (To a very large degree)

#### Sensitivity analyses

The Fischer’s exact test showed a significant difference between the two groups in the proportion of absolute agreement on whether the proposal was eligible for the funding programme, with the general feedback group demonstrating a higher rate of eligibility agreement (*p* < .01) in the 2018 review.

The proportion of absolute agreement on proposal score, defined as a difference in score of 0 or 1, was 49.7 and 49.6% in the general and individual feedback groups respectively (*p* = 1.000), supporting the findings of the main analysis of average absolute differences.

The multilevel model where absolute score differences were nested within reviewers instead of within proposals gave almost identical results as the model where absolute differences were nested within proposals (Appendix Table 6).

#### Overall call agreement and scoring

As this study present real funder data, regardless of the interventions, we present here the overall ICC for all proposals reviewed in 2017 and 2018. In line with the significant decrease in absolute score difference, there was an increase in overall ICC (1, 3) from 0.334 in 2017 to 0.428 in 2018 (Appendix Fig. 8). For comparison, ICC (1, 3) for the other calls of the funder can be seen at bit.ly/DamICC.

## Discussion

The results of this study suggest that proposal score agreement between reviewers who received individual feedback reports did not differ from that of reviewers who received a general feedback report. In addition, we observed higher eligibility agreement in the general feedback group in 2018.

To our knowledge, the present study is the first to evaluate the effect of training interventions in grant peer review in a real-world setting. One previous controlled study investigated the effect of reviewer training in grant peer review and found that interrater reliability was significantly improved in the training group [12]. The training applied in the previous study was an 11-min training video focusing on the general importance of the review and “how to assign evaluation scores”. Hence, both the training intervention and the review task differed from our study.

Furthermore, journal peer review has several similarities to the review of grant proposals, and research regarding training for journal review may also be relevant for funders. In a systematic review, Bruce et al. [11] found that training interventions to improve peer review in biomedical journals had a limited effect on the quality of the review report as assessed by journal editors.

The lack of effect of individual feedback compared to general feedback on scoring agreement may be related to the content and the simplicity of the individual feedback reports. The part of the individual feedback report addressing the reviewers’ scoring history provided the reviewers with information on their average score and their score distribution compared to the committee as a whole. It did not provide specific information on how the reviewers should re-score the proposals, interpret the content of the proposals or interpret and weigh the different criteria. The results suggest that merely adjusting the scores to align better with the average distribution of scores might increase agreement somewhat, but likely not by much. Supplementation of the individual feedback report with more comprehensive guidance on interpretation and possible actions could have been beneficial. Nevertheless, an automatically generated feedback report is probably not suitable for providing such specific guidance.

In the part of the individual feedback report addressing eligibility, reviewers who had rated many proposals as non-eligible were advised to reconsider the number of non-eligible proposals. This guidance did not increase the proportion of reviews assessing the proposal as eligible or the proportion of agreement on eligibility in the individual feedback group. However, in the general feedback group, for which no information on eligibility was provided, there was a significant increase in the proportion of agreement on eligibility compared to the individual feedback group. We have no plausible explanation for this difference and suspect this is an artefact.

### Limitations

One potential limitation of the study is that the score distribution provided in the general feedback report might have affected the reviewers in this group in a similar way as the reviewers who received the individual feedback report. A group that was offered no feedback would have provided a comparison with the usual process but would remove any possibility for blinding the participants (as was the intention with providing the general feedback). Furthermore, the group of reviewers were a heterogeneous sample, comprising reviewers of both scientific and non-scientific backgrounds, different ages and different levels of both reviewer and academic experience. Combined with the limited number of reviewers, this means that we cannot rule out any potential beneficial effects of the individual feedback report in a study with sufficient power.

Additionally, the two groups reviewed different sets of proposals. Even though, we do not suspect systematic differences influencing the agreement levels, we cannot rule this out and a RCT with two groups assessing the same proposals would be preferrable.

## Conclusions

Several factors may influence and decrease agreement between raters. Theoretically, training interventions have the potential to make reviewers focus on and value the same aspects of a proposal and to interpret and use the review scoring instrument more uniformly. However, to accomplish this, the training intervention should also focus on how to interpret the review criteria and address other factors that are associated with low agreement levels in the specific setting in which they are intended to be used.

Considering this and to ensure compliance and uniform interpretation of the report, a separate training session with the reviewers might be necessary. The feedback report combined with a training session would, however, be a more complex intervention and would have to be tested in a separate study.

Despite the lack of effect of the individual feedback report compared to the general feedback report on scoring agreement, the individual feedback report might still be considered useful. There was a significant decrease in absolute score difference in 2018 compared to 2017. This is supported by the increase in overall ICC (1, 3) for all three reviews for all submitted proposals. There might be causes other than the reports for this increase (e.g., more reviewer experience). However, given this increase and the fact that the intervention can be provided using a simple spreadsheet template and that the reviewers perceived the feedback report as useful, it might be reasonable to provide the report.

Even with the increase in agreement from 2017 to 2018, agreement levels were still critically low. Previous studies and reports have shown that this is a major concern across different programmes, funders and journals [2, 4, 8]. Increasing levels of agreement should be a priority of funders, primarily to increase consistency in the decision-making process. Given the agreement levels reported in this and similar studies, most funders likely use too few reviewers to compensate for the low agreement and achieve reasonable consistency. Hence, increasing the number of reviewers is still crucial to ensure acceptable overall levels of reliability of the funding process. Research to identify factors contributing to this phenomenon as well as the development and testing of interventions to increase agreement rates are still needed. Furthermore, an attempt should be made to justify costly peer review processes by evaluating their validity and explore alternative ways of allocating funds.

## Supplementary Information

**Additional file 1.** Appendix 1: Supplementary information.

## Data Availability

Data and materials were uploaded to and freely available at OSF.io/6rdvc under the CC BY-NC licence (creativecommons.org/licenses/by-nc/4.0).

## Abbreviations

ICC: Intra-class correlation
HRCS: Health research classification system
NOK: Norwegian krone
AD: Average deviation
CI: Confidence interval
AC1: Gwet’s agreement coefficient 1
LMM: Linear mixed model
