# Supplementary material for: Individual versus general structured feedback to improve agreement in grant peer review: a randomized controlled trial
Source: Res Integr Peer Rev. 2021 Sep 30;6:12. doi: 10.1186/s41073-021-00115-5 (PMC8485516; doi:10.1186/s41073-021-00115-5)
Supplement: Supplementary file 1 — Additional file 1. Appendix 1: Supplementary information. [file 41073_2021_115_MOESM1_ESM.docx]

Appendix 1: Supplementary information

Individual versus general structured feedback to improve agreement in grant peer review: A randomized controlled trial

Jan-Ole Hesselberg; Knut Inge Fostervold; Pål Ulleberg; Ida Svege

Research Integrity and Peer Review

Table of Contents

[Figures 2](#_Toc80711057)

[Figure 1. Distribution of scores in 2017 and 2018 2](#_Toc80711058)

[Figure 2. Distribution of absolute score difference in 2017 2](#_Toc80711059)

[Figure 3: Distribution of absolute score difference in 2018 3](#_Toc80711060)

[Figure 4. Average absolute score difference per reviewer in the individual feedback group at baseline and follow-up 4](#_Toc80711061)

[Figure 5. Average absolute score difference per reviewer in the general feedback group at baseline and follow-up 5](#_Toc80711062)

[Figure 6. Average proposal score per reviewer in the individual feedback group at baseline and follow-up 7](#_Toc80711063)

[Figure 7. Average proposal score per reviewer in the general feedback group at baseline and follow-up 8](#_Toc80711064)

[Figure 8. Intraclass correlation coefficient, one-way random effects, for all three reviewers in 2017 and 2018 9](#_Toc80711065)

[Tables 9](#_Toc80711066)

[Table 2. Eligibility agreement in general feedback group in 2017 11](#_Toc80711067)

[Table 3: Eligibility agreement in individual feedback group in 2017 11](#_Toc80711068)

[Table 4. Eligibility agreement in general feedback group in 2018 12](#_Toc80711069)

[Table 5. Eligibility agreement in individual feedback group in 2018 12](#_Toc80711070)

[Table 6. Linear mixed regression model analysis estimating the change in differences score over time by group. 4076 difference scores nested within 43 reviewers 13](#_Toc80711071)

# Figures

##

## **Figure 1.** Distribution of scores in 2017 and 2018


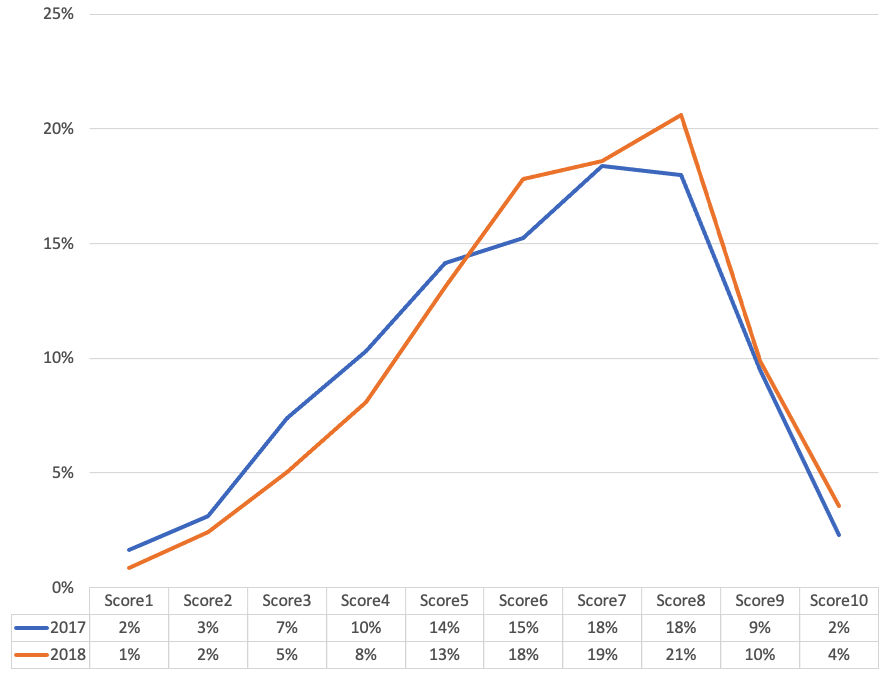


## **Figure 2.** Distribution of absolute score difference in 2017


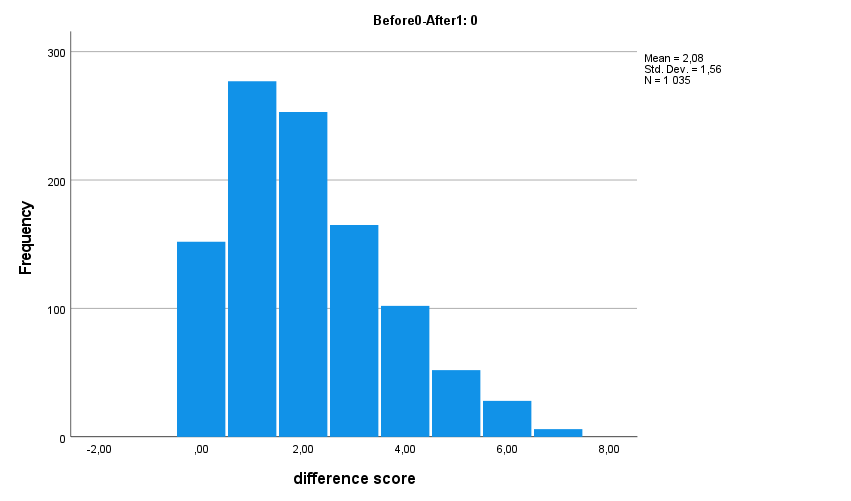


## **Figure 3:** Distribution of absolute score difference in 2018


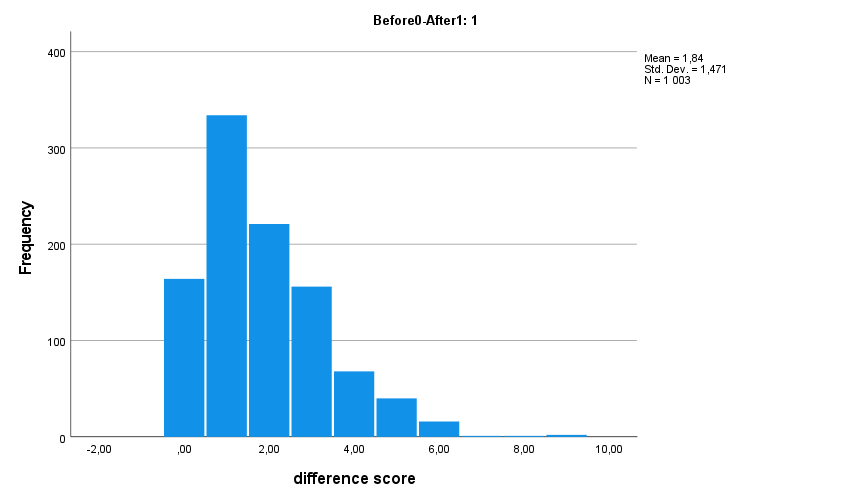


## **Figure 4**. Average absolute score difference per reviewer in the individual feedback group at baseline and follow-up


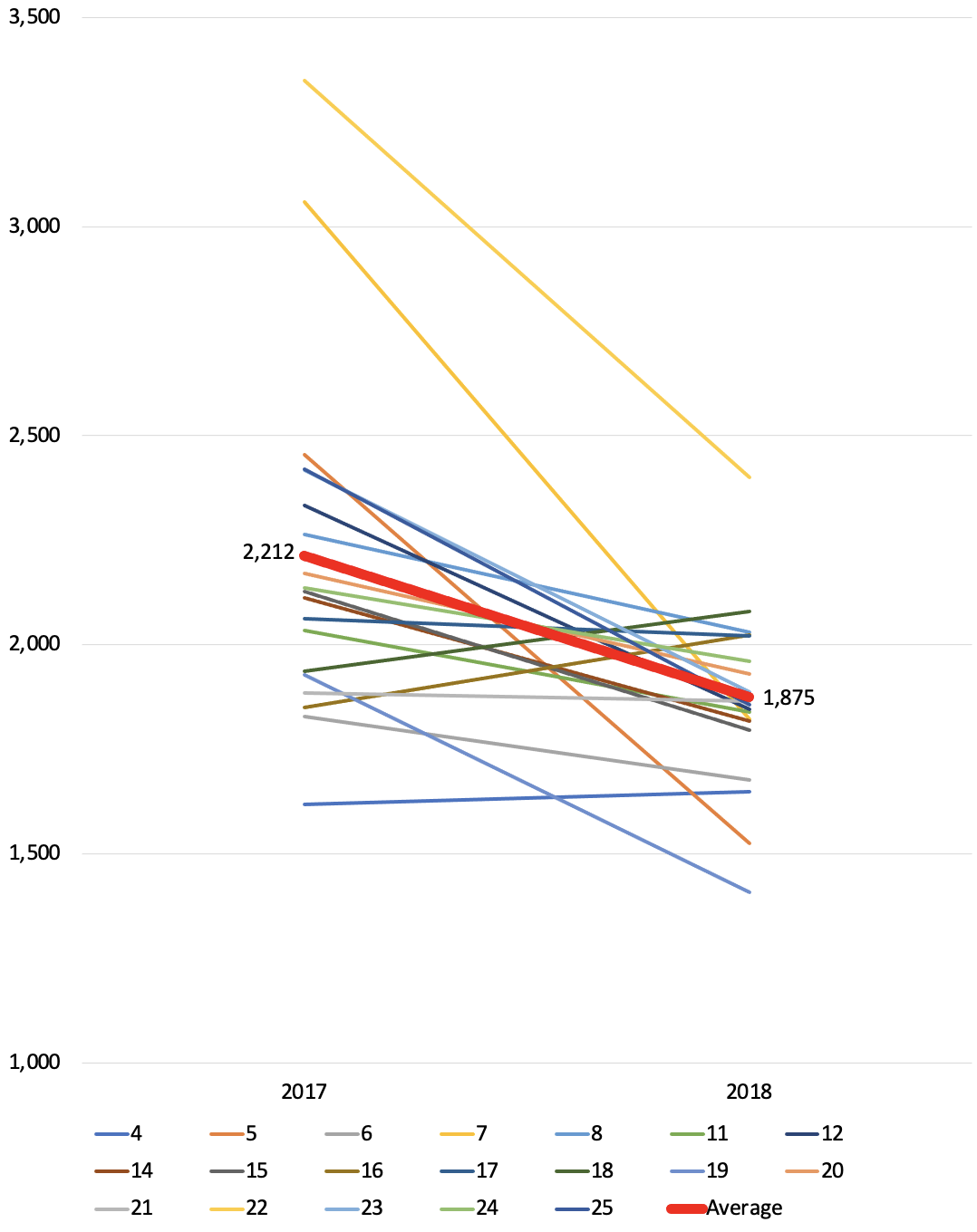


## **Figure 5**. Average absolute score difference per reviewer in the general feedback group at baseline and follow-up


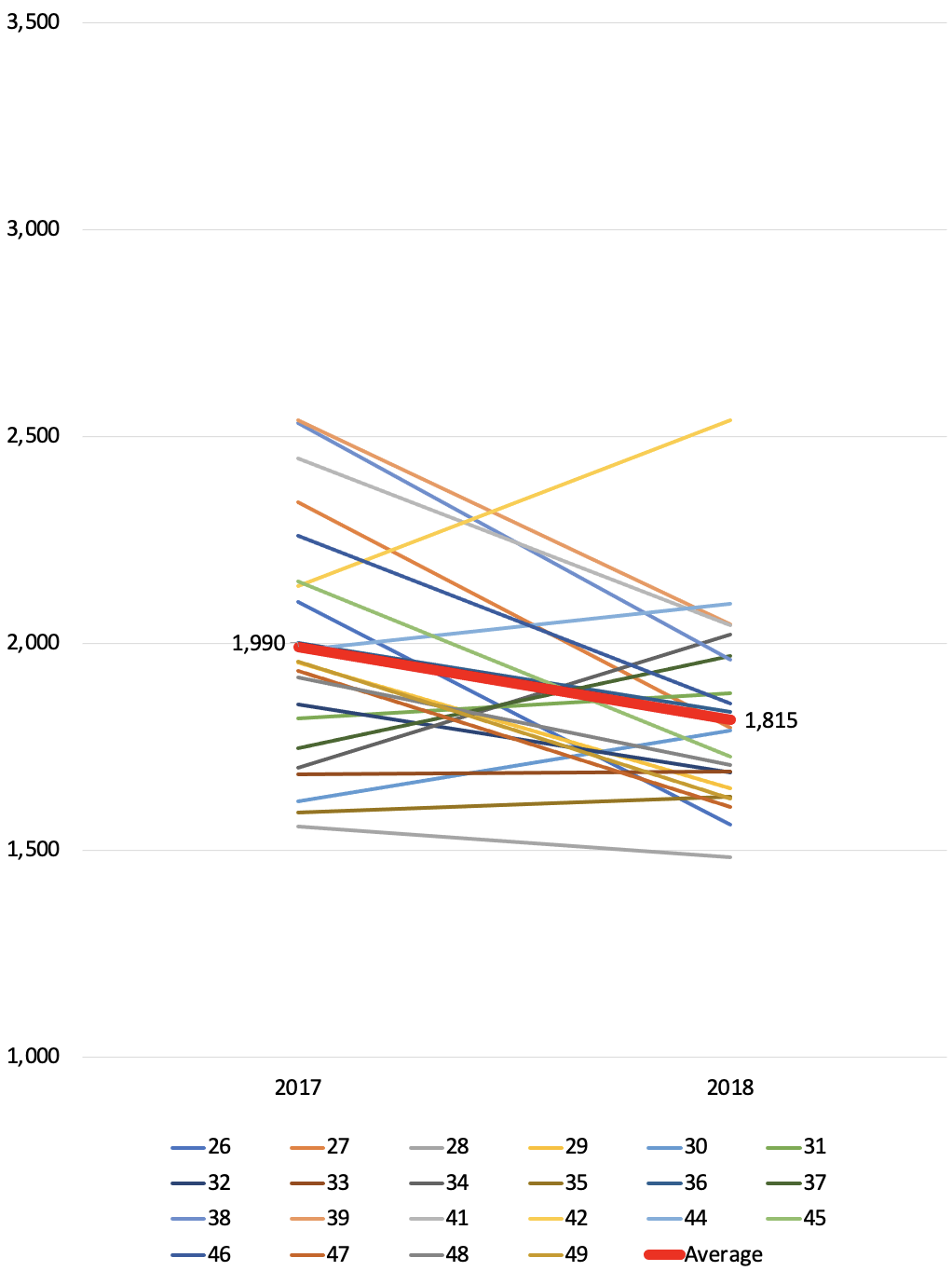


## **Figure 6.** Average proposal score per reviewer in the individual feedback group at baseline and follow-up


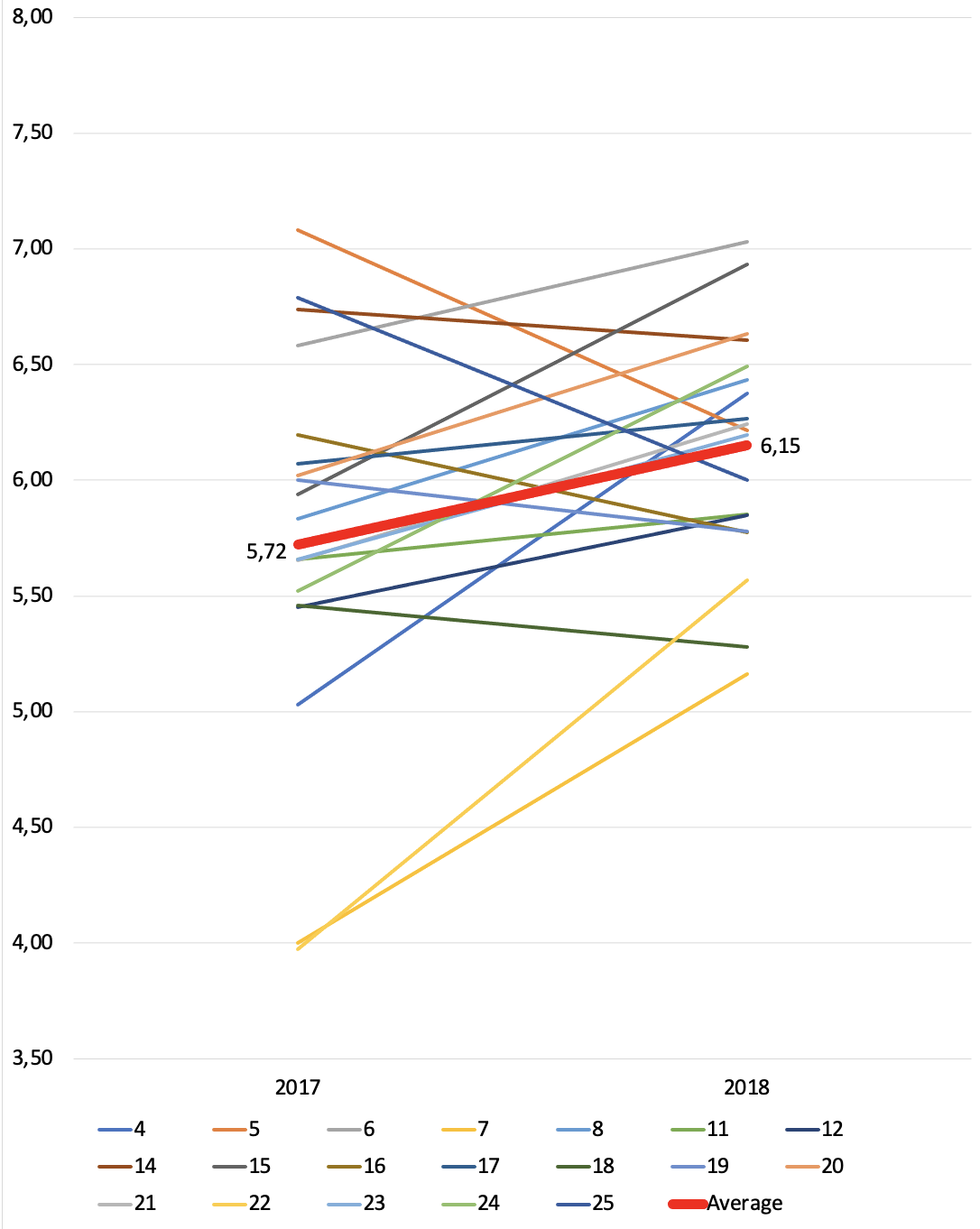


## **Figure 7.** Average proposal score per reviewer in the general feedback group at baseline and follow-up


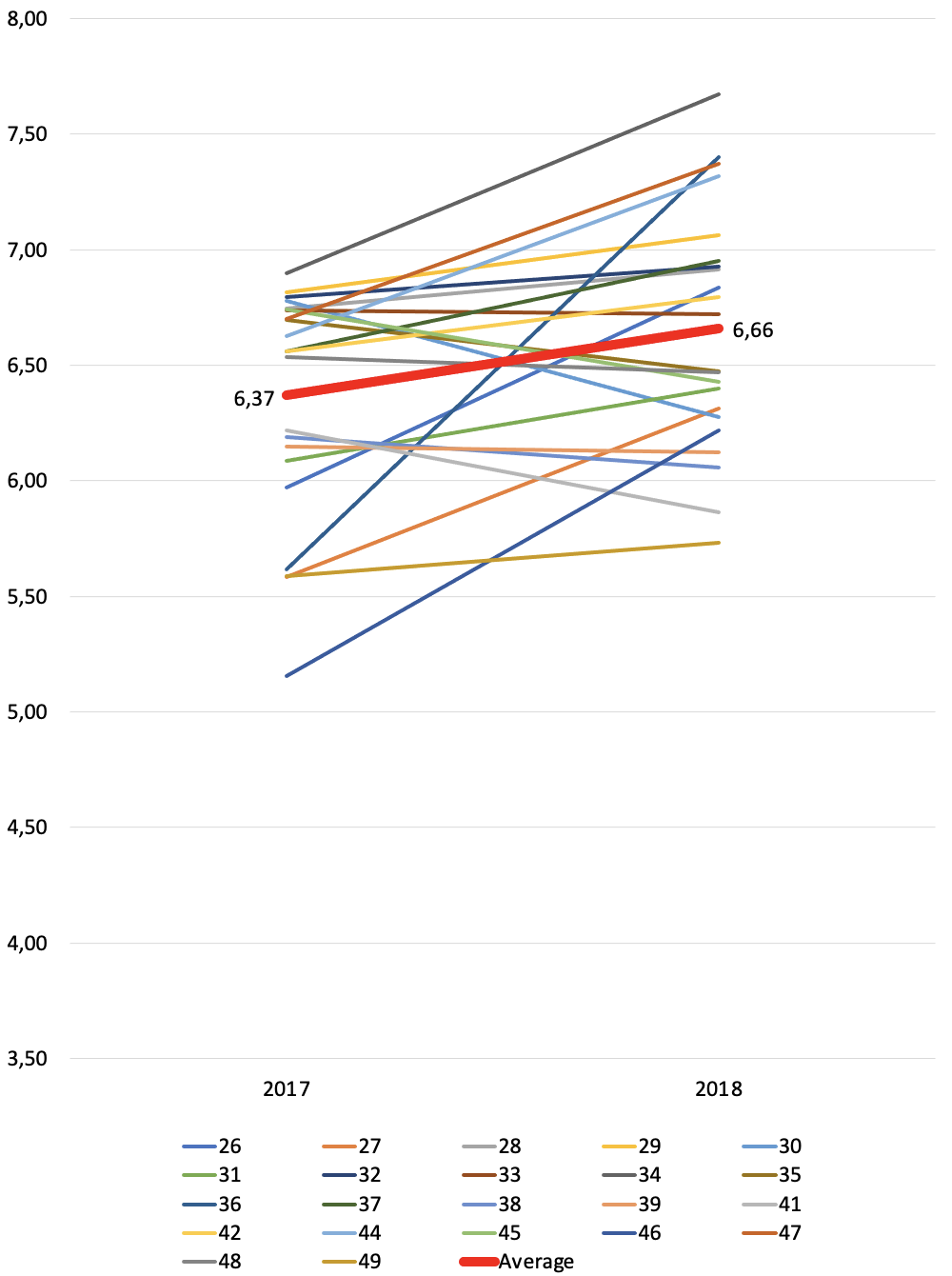


## **Figure 8.** Intraclass correlation coefficient, one-way random effects, for all three reviewers in 2017 and 2018


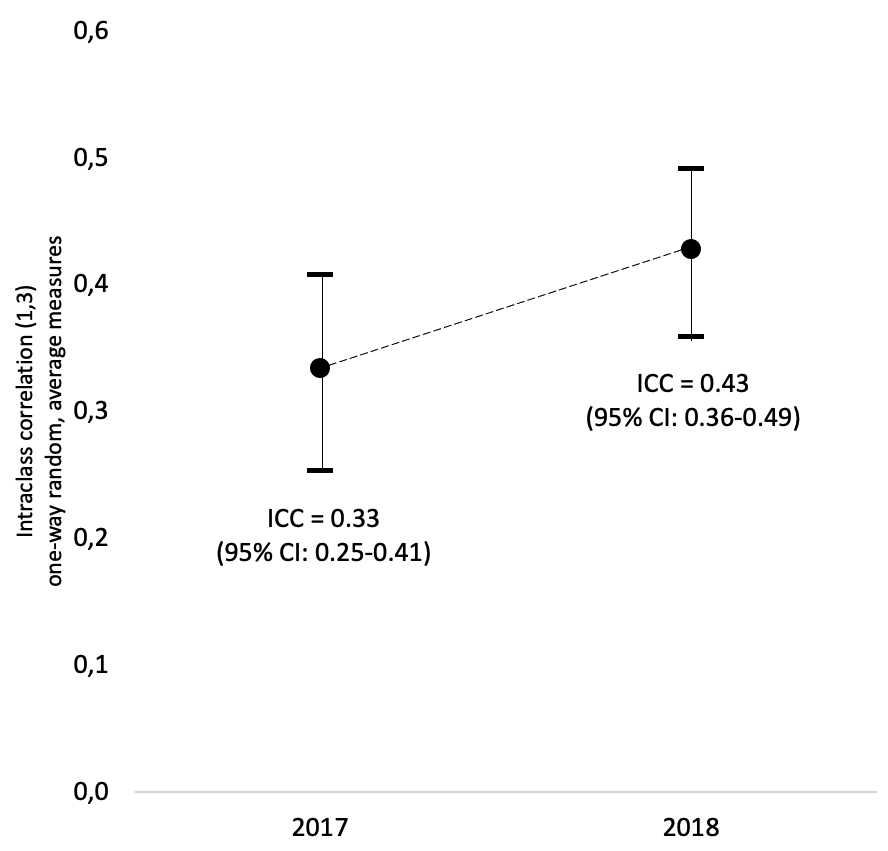


# Tables

**Table 1.** Reviews concluding with the proposal being eligible

| Number of reviews per proposal that concluded with the proposal being eligible | 2017 | 2018 |
| --- | --- | --- |
| 0 | 11 (0,9 %) | 6 (0,6 %) |
| 1 | 72 (6,0 %) | 36 (3,5 %) |
| 2 | 255 (21,3 %) | 135 (12,9 %) |
| 3 | 859 (71,7 %) | 867 (82,9 %) |

**Table 2.** Eligibility agreement in general feedback group in 2017

**
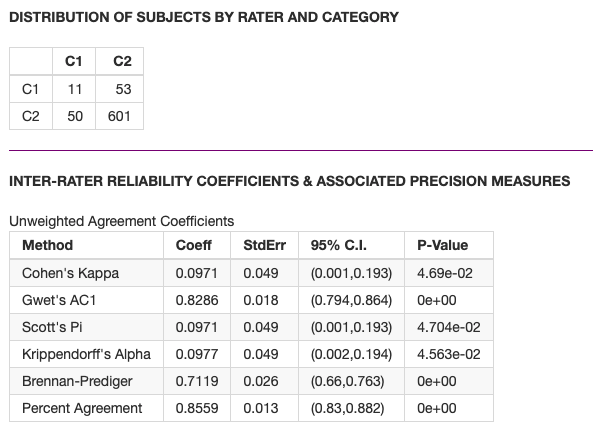
**

## **Table 3:** Eligibility agreement in individual feedback group in 2017

**
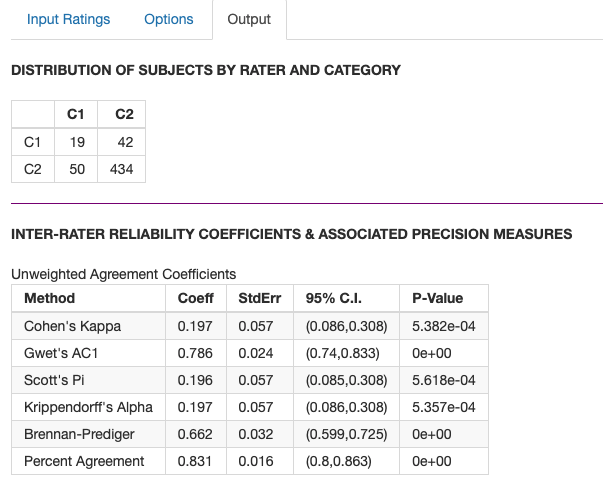
**

**Table 4.** Eligibility agreement in general feedback group in 2018

**
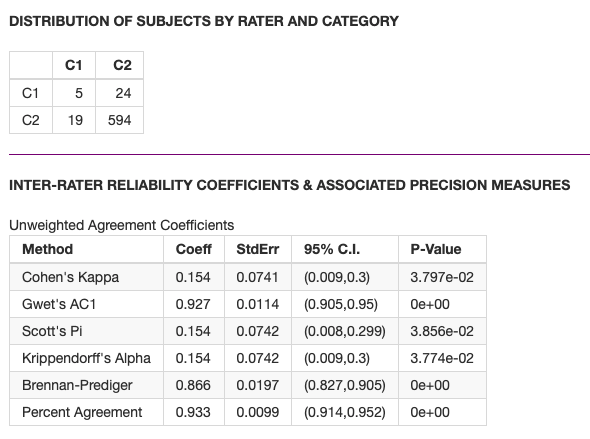
**

## **Table 5.** Eligibility agreement in individual feedback group in 2018

**
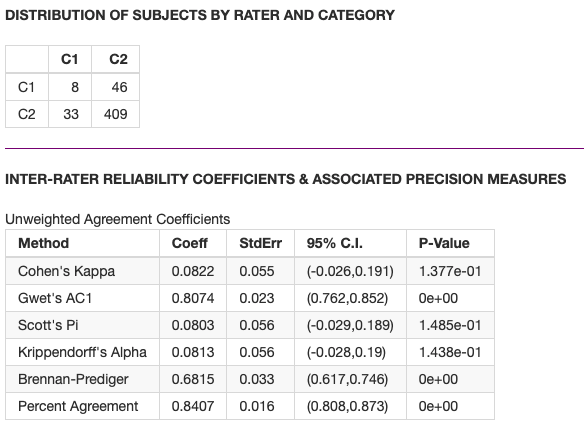
**

**Table 6.** Linear mixed regression model analysis estimating the change in differences score over time by group. 4076 difference scores nested within 43 reviewers

|  | *b* (*se*) | *t*-value | *p* | 95 % CI |
| --- | --- | --- | --- | --- |
| Intercept | 2.21 (0.07) | 78.545 | <.001 | [2.08, 2.32] |
| Time*^a^* | -0.34 (0.07) | -4.53 | <.001 | [-0.50, -0.14] |
| Group*^b^* | -0.22 (0.09) | -2.313 | .024 | [-0.38, -0.11] |
| Time×Group | 0.17 (0.10) | 1.735 | .083 | [-0.05, 0.42] |

^a^Baseline=0, follow-up=1, ^b^Individual feedback group=0, General feedback group=1
